# A New Natural Antioxidant Biomaterial from *Cinnamomum osmophloeum* Kanehira Leaves Represses Melanogenesis and Protects against DNA Damage

**DOI:** 10.3390/antiox8100474

**Published:** 2019-10-11

**Authors:** Yung-Shu Ho, Jane-Yii Wu, Chi-Yue Chang

**Affiliations:** 1 Department of Food Science and Biotechnology, No.168, University Rd., Da-Yeh University, Dacun, Chang hua 51591, Taiwan; verysmartme@gmail.com; 2 Department of Health Food, Chung Chou University of Science and Technology, No. 6, Lane 2, Sec. 3, Shanjiao Rd., Yuanlin Township, Chang hua 51591, Taiwan; charles561219@gmail.com

**Keywords:** antioxidant activity, *Cinnamomoum osmophloeum*, hydrosol, tyrosinase inhibitory activity, whitening, DNA damage protective effects

## Abstract

*Cinnamomoum osmophloeum* Kanehira (COK) is an indigenous tree species in Taiwan. Chemical compositions, antioxidant activity, mushroom tyrosinase inhibition, melanin synthesis repression, and protection against DNA damage of hydrosol from the COK leaves by steam distillation were examined. We performed 1,1-diphenyl-2-picrylhydrazyl radical scavenging, metal ion chelating, reducing power, and Trolox equivalent antioxidant capacity (TEAC) assays and determined the correlations between total phenolic contents and antioxidant activities. The findings showed that the anti-oxidative properties of COK hydrosol are closely correlated with their phenol contents. Additionally, the major constituents of hydrosol, i.e., cinnamaldehyde and benzaldehyde, had dose-dependent anti-tyrosinase effects against both monophenolase and diphenolase activities. GC-MS analysis revealed that the major bioactive components of hydrosol were trans-cinnamaldehyde (87.7%), benzaldehyde (7.0%), and cinnamyl acetate (5.3%). Moreover, we found that the hydrosol with the presence of benzaldehyde is more potent than pure cinnamaldehyde, and enhances the tyrosinase inhibitory activity of hydrosol. In kinetic analyses, Lineweaver–Burk plots and replots showed that COK hydrosol is a mixed-type inhibitor. Additionally, we found that very low doses of COK hydrosol repressed α-melanocyte-stimulating hormone-induced synthesis of microphthalmia-associated transcription factor, leading to decreased melanin synthesis in B16-F10 melanoma cells. These results demonstrated that production of hydrosol from COK leaves using steam distillation may provide a safe and efficacious source of skin-whitening agents for cosmetic and pharmaceutical applications, with antioxidant, anti-tyrosinase, anti-melanogenesis, and DNA protective activities.

## 1. Introduction

Human skin disorders such as lentigo, age spots, melasma, and malignant melanomas have been linked to melanin formation and accumulation [1]. Inhibition of tyrosinases and melanogenesis can ameliorate dermatological disorders and hyperpigmentation syndrome. Additionally, the global market for skin-whitening agents and cosmeceutical products has expanded recently because lighter skin color is preferred by many darker-skinned individuals [2]. Therefore, effective and safe tyrosinase and melanin synthesis inhibitors are considered important for the prevention of pigmentation illness and other melanin-related human health issues [3,4]. Additionally, environmental toxicants cause various oxidative stresses to human beings, and can produce reactive nitrogen species or reactive oxygen species (ROS) [5]. Excessive free radicals and ROS are associated with inflammatory signaling pathways, which eventually lead to cellular damage, aging, neural disorders, diabetes, atherosclerosis, inflammatory, cardiovascular disease, cancer, and melanogenesis [6,7]. Several studies suggest that antioxidants can inhibit the oxidative pressure damages by acting as free radical scavengers or ROS scavengers [7,8]. Moreover, both inhibitors of ROS generation and ROS scavengers may reduce melanin pigments production [9]. Specifically, tyrosinase is a key enzyme that catalyzes a rate-limiting step at the first two steps during the melanin synthesis processes [10], and down-regulation of tyrosinase is a critical goal for the prevention of skin illness and the production of skin-whitening agents [11]. Therefore, the development of natural antioxidants that effectively inhibit tyrosinases for the prevention or treatment of undesirable skin hyperpigmentation and protect against DNA damage is essential.

*Cinnamomoum osmophloeum* Kanehira (COK) is a Taiwanese indigenous cinnamon species with many uses as a Chinese herbal medicine. Active compounds of COK essential oils show excellent potential as pharmacological antibacterials [12]. Although previous studies of alcohol extracts of COK leaves demonstrated anti-tyrosinase activities, almost all of these studies focussed on ethanol extracts or essential oils from COK. Moreover, no quantitative and systematic studies have reported antioxidant properties, tyrosinase inhibitory activities and repression of melanin synthesis by water extracts (hydrosol) of COK leaves. Therefore, this study was conducted to investigate the effects of COK hydrosol on oxidative stress and melanogenesis in B16F10 melanoma cells and protect against DNA damage. This study is the first detailed report of the chemical composition and antioxidant, tyrosinase inhibition, melanogenesis repressive, and DNA damage protective activities of hydrosol from COK leaves.

## 2. Materials and Methods

### 2.1. Chemicals and Antibodies

Mushroom tyrosinase, butylated hydroxytoluene (BHT), α-tocopherol, 6-hydroxy-2,5,7,8-tetramethylcroman-2-carboxylic acid (Trolox), trichloroacetic acid (TCA), gallic acid, potassium ferricyanide, ferric chloride, ferrous chloride, ferrozine, 1,1-diphenyl-2-picrylhydrazyl (DPPH), 2,2′-azino-bis-3-ethylbenzthiazoline-6- sulphonic acid (ABTS), Folin–Ciocalteu reagent, *L*-tyrosine, and *L*-3,4-dihydroxyphenylalanine (*L*-DOPA) were obtained from Merck Co. (Darmstadt, Germany). All the chemicals and solvents used in the study were of analytical grade or of high-performance liquid chromatography. Antibodies against microphthalmia-associated transcription factor (MITF) and β-actin were obtained from Cell Signalling Technology (Danvers, MA, U.S.A.) and Sigma Chemical Co. (St. Louis, U.S.A.), respectively.

### 2.2. Hydrosol Preparation: Extraction from COK Leaves Using Steam Distillation

**Figure 1:**
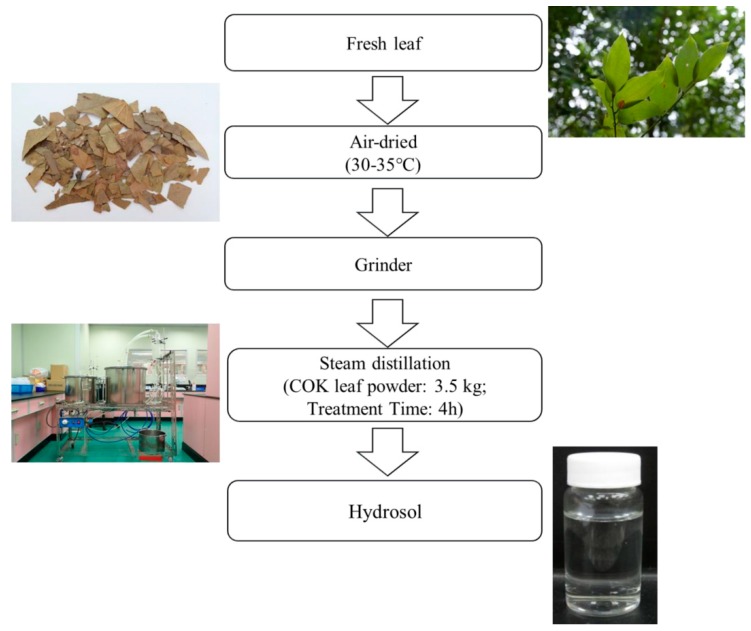
Process for producing hydrosol from *Cinnamomoum osmophloeum* Kanehira (COK) leaves using steam distillation.

Leaves from a 13-year-old COK tree in Taiwan (No.186, Yongping Road, Zhongliao Township, Nantou County, Taiwan) were air-dried. Using a stainless-steel grinder, the leaves were then ground into a fine powder (less than < 10 mesh) and were then stored at room temperature. Next, 3.5 kg of dried COK leaf powder was extracted using steam distillation for 4 h. This process was repeated four times. Using vacuum, the extract was filtered and then, using a rotary evaporator, dry extract was obtained (Figure 1). The extract was finally evaporated to obtain a final weight.

### 2.3. Gas Chromatography/mass Spectrometry (GC/MS) Analysis of Hydrosol

A Thermo-GC/MS instrument with a THERMO WaxMS cross-linked 5% phenyl- 95% methylpolysiloxane capillary column (60 m × 0.25 mm i.d., film thickness 0.25 μm) was used with helium as a carrier gas at a constant flow rate of 1.0 mL/min. The column was maintained at 200 °C for 5 min after injection and was then heated at 5 °C/min to 260 °C. Pure essential oil (1.0 mL) was injected with a split ratio of 1:100. The temperatures of the injector, transfer line and ion-source were 250 °C, 250 °C, and 200 °C, respectively. MS detection was performed in the electron impact mode at 70 eV ionisation energy and 60 μA ionisation current; the instrument was operated in full-scan acquisition mode in the range of 50–350 amu. Compounds were identified by comparing the retention times and indices of chromatographic peaks with those of authentic reference standards, injected under the same conditions. MS fragmentation patterns were compared with those of pure compounds, and the mass spectrum database were searched using the National Institute of Standards and Technology (NIST) MS spectral database [13].

### 2.4. Total Phenolic Content

Total phenolic contents (TPC) of the extracts were determined using the Folin–Ciocalteu assay as previously described [14]. Briefly, hydrosol samples (100 μL) were mixed with 100 μL aliquots of Folin–Ciocalteu reagent (10-fold dilution) and 10 μL aliquots of sodium carbonate (10%, *w/v*). After storage for 30 min, the absorbance was measured at 735 nm. TPCs are expressed as gallic acid equivalents (GAE) in mg per 100 g of fresh material.

### 2.5. DPPH Free Radical Scavenging Assays

As previously described, using DPPH assay, radical scavenging activities of hydrosol were determined [15], with modifications. Briefly, varying dilutions of hydrosol samples (75 μL in triplicate) were added to 150 μL aliquots of DPPH (0.02 g/100 mL) and absorbance was measured at 517 nm after 30 min. BHT was used as a positive control (0.5 mg/mL ethanol). Radical scavenging activities were expressed as inhibition ratios (%) using the following equation:

The inhibition ratio(%)=[1−(A/B)]×100%

where A is the absorbance of the hydrosol sample and B is the absorbance of the blank.

### 2.6. Fractional Inhibitory Concentrations

Assays of fractional inhibitory concentrations (FIC) were performed as described previously [16], with modifications. Briefly, FeSO_4_ (20 μL; 2 mM) was mixed with different dilutions of hydrosol samples (200 μL). After adding ferrozine (40 μL; 5 mM), reactions were allowed to proceed for 10 min and and then absorbance was measured at 562 nm. Furthermore, 0.5 mg/mL ethylene diamine tetra acetic acid (EDTA) was used as a positive control. Percentage inhibition of ferrozine-Fe^2+^ complex formation was calculated using the following formula:

Metal chelating effect (%)=[1−(A/B)]×100%

where A is the absorbance of the hydrosol sample and B is the absorbance of the blank.

### 2.7. Reducing Power Assays

The reducing power of COK hydrosol was determined by monitoring the reduction of Fe^3+^ to Fe^2+^ as described previously [17], with modifications. Briefly, to 50 μL dilutions of hydrosol, 50-μL aliquots of phosphate buffer (pH 6.6, 200 mM) and 50-μL aliquots of potassium ferricyanide (1%, *w/v*) were added. After incubation at 50 °C for 20 min, 50 μL of TCA (10%, *w/v*) was added; and the mixture was centrifuged at 9,000 rpm for 3 min. Finally, 50 μL supernatant was mixed with 50 μL distilled water and 50 μL ferric chloride in water (0.1%, *w/v*), and absorbance was measured at 700 nm against a blank after 10 min of reaction time. Positive controls used were butylated hydroxytoluene (BHT) and α-tocopherol.

### 2.8. Trolox Equivalent Antioxidant Capacity (TEAC) Assay

Antioxidant activities of COK hydrosol were assessed according to a previously described method [15,18], with modifications. Briefly, concentrated ABTS radical cation (ABTS•^+^) solution was diluted in phosphate buffered saline (pH 7.4) to a final absorbance of 0.80 ± 0.05 at 734 nm. After mixing (0.02 mL) sample with 1 mL ABTS^+^ solution, there were reductions in absorbance at 734 nm after 5 min. Trolox was used as a standard, and hydrosol activities were expressed as TEAC by plotting Trolox calibration curves. Molar equivalent TEAC values of hydrosol samples were calculated according to decreases in absorbance of Trolox solution. BHT and α-tocopherol were used as positive controls.

### 2.9. Effects of Hydrosol on Inhibition of Mushroom Tyrosinase

Mushroom tyrosinase activity was measured using spectrophotometric analysis as previously described, with modifications [19]. Briefly, 20 μL *L*-tyrosine or 20 μL *L*-DOPA in PBS (pH 6.8; 80 μL) and 80 μL PBS with or without test hydrosol were added into 96-well microplates, and then 20 μL mushroom tyrosinase (100 U/mL) was added. After mixing and incubation at 37 °C for 20 min, dopachrome production (or consumption) in the reaction mixture was determined at 475 nm absorbance. Kojic acid, which inhibits tyrosinase, was used as a positive control. The percentage inhibition of tyrosinase was calculated as follows:

$$\mathrm{Inhibition}\;\left(\%\right)\equiv \frac{\left(A-B\right)-\left(C-D\right)}{\left(A-B\right)}\times 100\%$$

where A indicates absorbance with enzyme in the absence of hydrosol sample, B indicates absorbance without enzyme or hydrosol sample, C indicates absorbance with enzyme, and hydrosol and D indicates absorbance without the enzyme but with hydrosol. We also calculated 50% tyrosinase inhibitory concentrations (IC_50_) of samples as those that inhibit tyrosinase activity by 50%.

### 2.10. Kinetic Analysis of Mushroom Tyrosinase Inhibition

Reaction mixtures contained 20 μL of *L*-tyrosine or *L*-DOPA (0.25 mg/mL) as substrates, 100 μL of mushroom tyrosinase (20 units/mL) in 0.2 M sodium phosphate buffer (pH 6.8) and 80 μL of each hydrosol sample in a total volume of 200 μL. The assay was performed at 25 °C, and inhibitory kinetics of each hydrosol sample with tyrosinase was analyzed using Lineweaver–Burk plots. The reciprocal equation was used for rapid equilibrium from the mixed-type noncompetitive inhibition, as expressed in Equation (1) [20]. *K_i_* values for hydrosol samples were estimated from slope replots (Equation (2)). The *αK_i_* values for hydrosol were calculated from the 1*/v* axis intercept replot (Equation (3)),

(1) $$\frac{1}{v}=\frac{K_{s}}{V_{max}}\left(1+\frac{\left[I\right]}{K_{i}}\right)\frac{1}{S}+\frac{1}{V_{max}}\left(1+\frac{\left[I\right]}{\alpha K_{i}}\right)$$

(2) $$Slope=\frac{K_{s}}{V_{max}}+\frac{K_{s}}{V_{max}K_{i}}\left[I\right]$$

(3) $$\frac{1}{v}\;axis\;intercept=\frac{1}{V_{max}\;\alpha K_{i}}\left[I\right]+\frac{1}{V_{max}}$$

where *V_max_* is the maximal velocity of the tyrosinase activity, *K_s_* is the dissociation constant of substrate (*S*) from the enzyme–substrate complex (ES), *K_i_* is the dissociation constant of inhibitor [I] from the enzyme-inhibitor complex and *αK_i_* is the dissociation constant of the inhibitor from the enzyme–substrate-inhibitor complex (*ESI*).

### 2.11. Cell Culture and Cell Viability Assays

B16-F10 melanoma cells (BCRC60031) were obtained from the Bioresource Collection and Research Center (BCRC), Taiwan. The B16-F10 cells were cultured in Dulbecco’s modified Eagle’s medium (Gibco, California, USA) containing 10% FBS at 37 °C in 5% CO_2_. By trypsinisation, cells were harvested. Cell viability was measured using 3-(4,5-dimethyl-thiazol-2-yl)-2,5-diphenyltetrazolium bromide (MTT) assay as described by Lee [15].

### 2.12. Cellular Melanin Contents

Melanin contents were measured using the method described by Lee. [15]. Briefly, B16-F10 cells were cultured in 6-well plates at a density of 0.8 × 10^5^ cells per well and were then incubated for 24 h. Cells were then treated with 100-nM α-melanocyte-stimulating hormone (α-MSH), kojic acid (positive control) and hydrosol at various concentrations for 24 h. After washing twice with PBS, cells were lysed in a buffer containing 100 mM sodium phosphate (pH 6.8), 1% Triton X-100 and 0.1 mM phenylmethane sulphonyl fluoride and stored at −80 °C for 30 min. After collecting the cells, cell pellet was dissolved in 1N NaOH containing 10% DMSO at 65 °C for 1 h. Thereafter, absorbance was measured at 405 nm.

### 2.13. Western Blot

Cells were lysed as described in Section 2.11 and subjected to Western blotting for MITF protein using an Anti-MITF antibody.

### 2.14. DNA Protection Assays

Oxidative DNA damage was determined according to the conversion of circular supercoiled pCI neo plasmid DNA into nicked circular or further degraded forms using a previously described method [21,22] with slight modifications. Reaction mixtures of 20 μL contained 2.5 μL of supercoiled *pCI neo* (150 ng/μL), 10 μL of a Fenton reaction solution containing 30 mM hydrogen peroxide, 100-μM ferric chloride and 100 μM ascorbic acid in 20 mM Tris-HCl buffer (pH 7.6), and 5μL of hydrosol (0.3325–5.32 mg/mL) or quercetin (positive control; 250 μg/mL). Reaction mixtures were incubated at 37 °C for 30 min and plasmid DNA forms were separated on 0.7% agarose gels and were stained using SafeView™ (Applied Biological Materials (ABM) Inc., Richmond, Canada).

To semi-quantify antioxidant activities of the extracts, quantities of supercoiled and nicked forms of *pCI neo* were quantified using an AlphaImager Mini (proteinsimple) instrument and band intensities on agarose gels were quantified using Gelpro software. As negative and positive controls, *pCI neo* plasmid was incubated alone and with the Fenton reagent mixture, respectively. Data are expressed as quantities of supercoiled DNA relative to that (100%) in negative controls. Protective activities of extracts were calculated from quantities of supercoiled and nicked plasmid DNA using the following equations [23]:

$$Protection\;of\;supercoiled\;plasmid\left(\%\right)=\frac{supercoiled\;form\;intensity}{pCI\;neo\;DNA\;band\;intensity}\;\times \;100$$

$$Protection\;of\;nicked\;plasmid\left(\%\right)=\frac{nicked\;form\;intensity}{pCI\;neo\;DNA\;band\;intensity}\;\times \;100$$

### 2.15. Statistical Analysis

All data are expressed as means ± standard deviation (SD). Differences between treatments were identified using Student’s t-test or ANOVA followed by Scheffe’s test. Differences were considered significant when *p* < 0.05.

## 3. Results and Discussion

### 3.1. Identification of Volatile Compounds in Hydrosol

**Figure 2:**
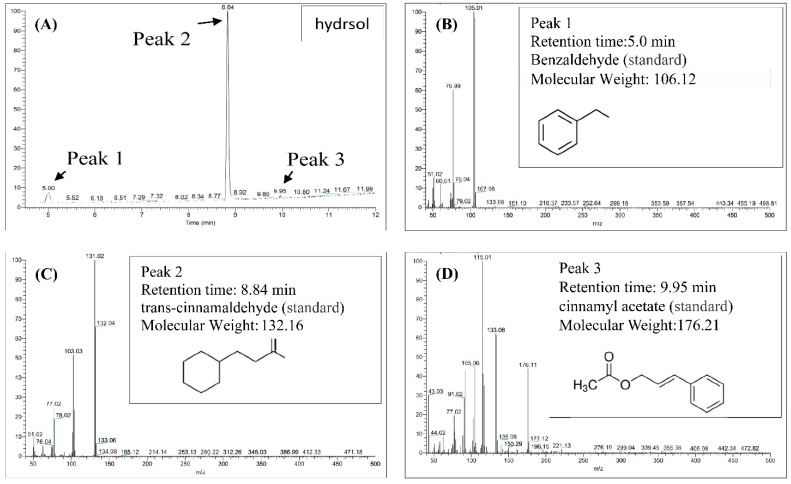
Gas chromatography/mass spectrometry (GC/MS) chromatograms of the major constituents of hydrosol from *Cinnamomoum osmophloeum* Kanehira (COK) leaves. (**A**) Hydrosol sample; Standard chemical: (**B**) benzaldehyde; (**C**) trans-cinnamaldehyde; (**D**)cinnamyl acetate.

Contents of hydrosols were analyzed using GC/MS. Components were identified according to retention times of standards and quantities were determined from peak areas of the eluents (Figure 2). As outlined in Figure 2A–D, trans-cinnamaldehyde, benzaldehyde, and cinnamyl acetate were the major compounds in COK hydrosol and were present at 87.7%, 7.0%, and 5.3%, respectively. Eugenol was previously found in essential oils from *C. verum* at 7.29% [24] but was not detected in the present COK hydrosol (Figure 2). Differences in extraction processes and assay methods may contribute to differences in cinnamaldehyde contents of *C. cassia* essential oils [25].

### 3.2. Antioxidant Properties of Hydrosol from COK Leaves

#### 3.2.1. DPPH Radical Scavenging Activity

**Figure 3:**
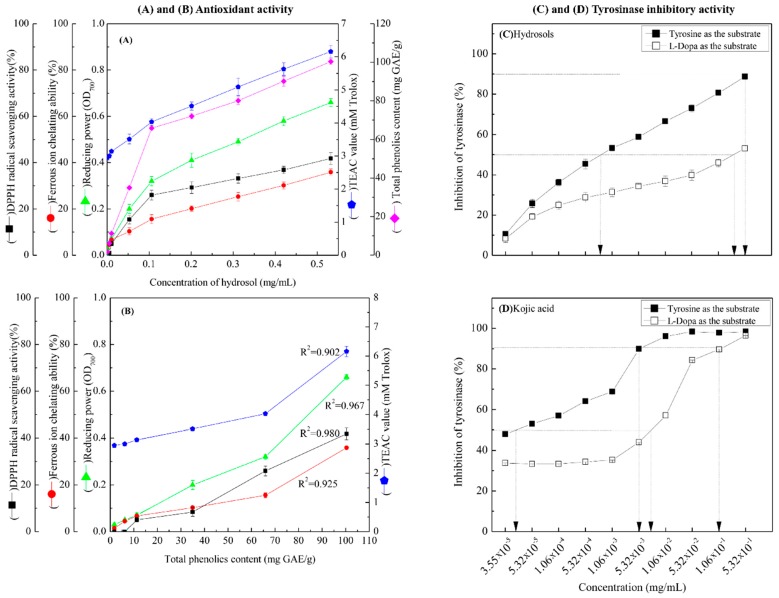
(**A**) Antioxidant activities of COK hydrosol produced by steam distillation; (**B**) overview of the relationships between total phenolic contents (TPCs) in hydrosol and antioxidant activities; (**C**,**D**) dose-dependent inhibition of monophenolase and diphenolase activities of tyrosinase using *L*-tyrosine and *L*-DOPA as substrates, respectively. Values represent means ± standard deviations (SD; *n* = 3.).

In Figure 3A, DPPH radical scavenging activities of hydrosols from 1.06 × 10^−2^ to 5.32 × 10^−1^ mg/mL were between 5.04% ± 0.96% and 41.76% ± 2.50%, respectively. These data indicate dose-dependent radical scavenging by the COK hydrosol. In comparison, the positive controls BHT and α-tocopherol scavenged 94.32% ± 0.08% and 93.82% ± 0.13% of DPPH reactive radicals, respectively, at 0.5 mg/mL. The radical scavenging activity of COK hydrosol is most likely due to cinnamaldehyde (Figure 2). Many studies show that phenolics and flavonoids prevent the formation of DPPH radicals by donating hydrogen atoms. Hence, the DPPH radical scavenging activities of *Cinnamomum* species may also reflect actions as a hydrogen donor [26,27].

#### 3.2.2. Metal Ion Chelation

We assessed the Fe^2+^ binding capacity of hydrosols at 1.06 × 10^−3^ and 5.32 × 10^−1^ mg/mL, as shown in Figure 3A. Metal chelation by hydrosol components marginally increased with concentration, but at 6 mg/mL, had only 37.3% of the chelating activity of EDTA (Figure 3A).

#### 3.2.3. Reducing Power

As shown in Figure 3A, the reducing power of hydrosol components was concentration-dependent and was higher than those of BHT and α-tocopherol (0.22 ± 0.01 and 0.33 ± 0.15, respectively) at 0.5 mg/mL. In previous studies, the reducing power of crude extracts from *C. osmophloeum* twigs in BuOH fractions were the highest among all fractions and increased linearly with concentration [28]. Because antioxidant potentials differ between compounds in extracts, overall antioxidant activities of extracts strongly depend on the extraction solvent. However, the reducing power (OD value = 0.7) of water extracts from *C. osmophloeum* twigs was similar (OD value = 0.66 ± 0.01) to that of the present *C. osmophloeum* hydrosols (Figure 3A), which were produced using steam distillation. Reducing power is generally associated with the presence of reducing agents and antioxidant activity, reflecting cessation of free radical chain reactions by the provision of reducing hydrogen atoms. The reducing power of *C. osmophloeum* is probably due to the presence of the hydroxyl groups in phenolic compounds (Figure 2), which might act as electron donors.

#### 3.2.4. TEAC Assays

TEAC values of the present hydrosol were increased at concentrations higher than 5.32 × 10^−3^ mg/mL and were greater than those of the reference compounds α-tocopherol and BHT, as shown in Figure 3A. Steric accessibility to the radical site of ABTS^+^ is the main criterion for activity in TEAC assays. Accordingly, the phenol groups of the present hydrosol constituents are slightly less hindered than that of Trolox.

#### 3.2.5. Correlations between TPCs and Antioxidant Activities

As shown in Figure 3B, there was a highly significant correlation between TPCs and DPPH free radical scavenging activity at GAE from 1.75 ± 0.438 to 100.41 ± 1.581 mg/g. Correlation coefficients of TPC with DPPH free radical scavenging activity, FIC activity, reducing power and TEAC were 0.980, 0.925, 0.967 and 0.902, respectively (Figure 3B). Collectively, these data demonstrate that the antioxidant activities of COK hydrosols are closely correlated with phenol contents.

### 3.3. Effect of COK Hydrosol Concentration on Tyrosinase Activity

We determined the effects of COK hydrosol concentration on the oxidation of *L*-tyrosine and *L*-DOPA by mushroom tyrosinase and made comparisons with the activity of kojic acid, which is a well-known tyrosinase inhibitor (Figure 3C,D). The present hydrosol potently and dose-dependently inhibited *L*-tyrosine and *L*-DOPA oxidase activities of mushroom tyrosinase (Figure 3C) but had higher tyrosinase inhibitory activity in the presence of *L*-tyrosine substrate (monophenolase activity) than *L*-DOPA (diphenolase activity). IC_50_ values of kojic acid against monophenolase and diphenolase activities were 4.0 × 10^−5^ and 7.8 × 10^−3^ mg/mL (Figure 3C), respectively, significantly more than those of the present hydrosol (7.96 × 10^−4^ mg/mL and 0.35 mg/mL, respectively; Figure 3D). Hence, to achieve 90% tyrosinase inhibitory activity (monophenolase activity), hydrosol and kojic acid were required at 0.52 mg/mL and 4.0 × 10^−3^ mg/mL, respectively, when *L*-tyrosine was used as the substrate (Figure 3C,D). Although tyrosinase inhibitory effects require higher concentrations of COK hydrosol than kojic acid, potent tyrosinase inhibitory activity was evident in these experiments.

Various tyrosinase preparations, activity assay methods, and inhibitor purities and components could have contributed to the differences in enzyme inhibitory kinetics [29]. However, biological activities of the plant extracts are contributed by their bioactive components, which may be affected by the plant species, harvest time, season, geographical origin, agronomic practices, and extraction methods. Inhibitors have been identified and characterized from natural sources in many recent studies, and relationships between inhibitory activities and natural ingredients are well established (Table 1). In particular, many aldehydes and other compounds have been isolated and characterized as tyrosinase inhibitors. These include cinnamaldehyde, 2-hydroxy-4-methoxybenzaldehyde, anisaldehyde, cuminaldehyde, cumic acid, flavonols, flavones, and isoflavans [4,19,30,31,32,33,34,35,36,37]. The present hydrosol comprised 87.7% cinnamaldehyde and 7.0% benzaldehyde, suggesting that cinnamaldehyde, followed by benzaldehyde, is the main substance responsible for the tyrosinase inhibitory activity of hydrosol. In line with Table 1, we found that the hydrosol with the presence of benzaldehyde is more potent than pure cinnamaldehyde, and enhances the tyrosinase inhibitory activity of hydrosol. Benzaldehydes were also confirmed to inhibit both diphenolase activity and monophenolase activity of mushroom tyrosinase [38]. The tyrosinase inhibitory mechanism of benzaldehyde-type inhibitors presumably comes from their ability to form a Schiff base with a primary amino group in the enzyme [33,39]. The addition of an electron-donating group at the para position of benzaldehyde increases tyrosinase inhibitory activity, probably stabilizing the Schiff base.

### 3.4. Kinetic Modes of Mushroom Tyrosinase Inhibition by COK Hydrosol

**Figure 4:**
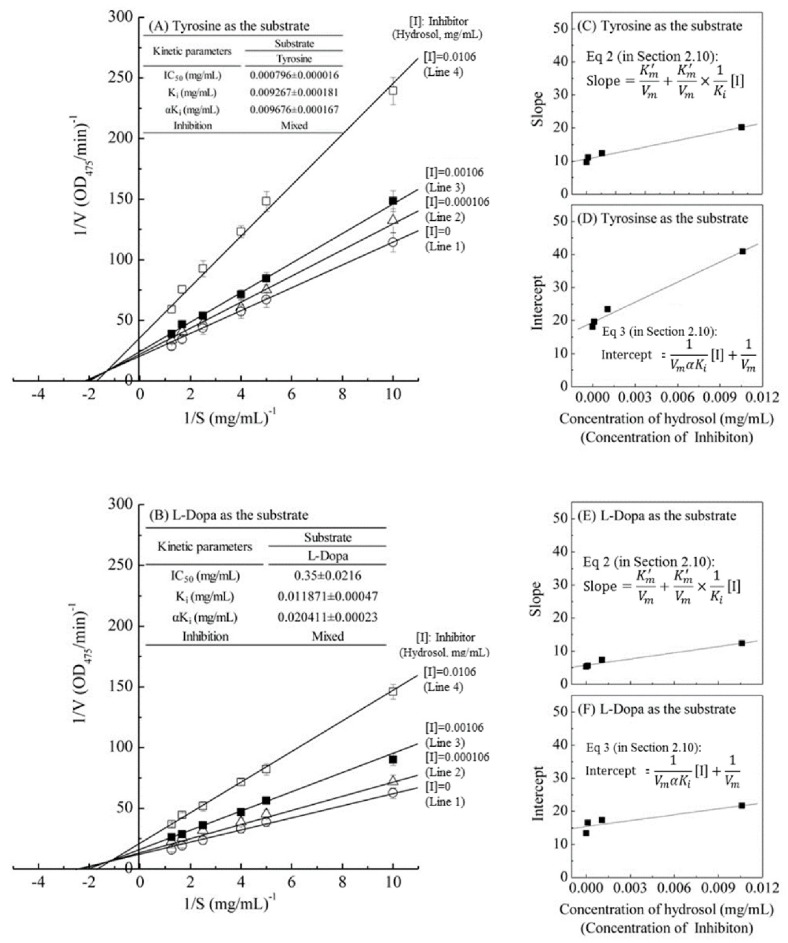
Lineweaver–Burk plots of L-tyrosine and L-DOPA catalysis by mushroom tyrosinase in the presence of inhibitor (hydrosol) at various concentrations. Concentration of inhibitor (hydrosol) for line 1–4 were 0, 0.000106, 0.00106, and 0.0106 mg/mL, respectively; (**A**) L-tyrosine substrate; (**B**) L-DOPA substrate; *insets* in (**C**,**E**) and (**D**,**F**) show secondary plots of slopes and intercepts of the straight lines versus inhibitor (hydrosols) concentrations, indicating *K_i_* and *αK_i_* values for L-tyrosine and L-DOPA respectively; *K_i_*, dissociation constant for inhibitor binding with free enzyme; *αK_i_*, dissociation constant for inhibitor binding with the enzyme-substrate complex. Equations (2) and (3) are detailed in Section 2.10.

The kinetic behaviors of hydrosol, on the monophenolase and the diphenolase activity of tyrosinase using *L*-tyrosine and *L*-DOPA as the substrate were studied, respectively. The tyrosinase inhibitory kinetics of hydrosol were analyzed using Lineweaver–Burk double-reciprocal plots, as shown in Figure 4. In Figure 4A,B, the four lines represent uninhibited enzyme (Line 1) and inhibition by three concentrations (Line 2 – Line 4) of hydrosol, and these lines intersect to the left of the 1*/v* axis above the *1/S* axis. Under the uninhibited experimental conditions, the maximum velocity (*V_max_*) of the *L*-tyrosine and *L*-DOPA oxidation reaction catalyzed by the tyrosinase were 0.055 ΔOD_475_/min and 0.096 ΔOD_475_/min without containing hydrosol (Line 1 in Figure 4A,B), respectively. The kinetic parameters *K_m_* (Michaelis constant) for mushroom tyrosinase obtained from a Lineweaver–Burk plot using *L*-tyrosine and *L*-DOPA as the substrates, respectively, show that *K_m_* was 0.534 mg/mL and 0.511 mg/mL without containing hydrosol (Line 1 in Figure 4A,B).

The four lines, obtained from the uninhibited enzyme and from three various concentrations of hydrosol, intersected to the left of the 1/v axis above the 1/S axis. Increased concentrations of hydrosol resulted in decreased *V_max_* and an increased *K_m_*. Specifically, when *L*-tyrosine was used as the substrate, increasing concentrations of hydrosol resulted in multiple lines with various slopes and intercepts, but these intersected each other in the second quadrant (Line 1–4 in Figure 4A). Similar results were obtained when *L*-DOPA was used as the substrate (Line 1–4 in Figure 4B), supporting the assertion that hydrosol carries mixed-type inhibitors of tyrosinase. In accordance with mixed-type inhibition, hydrosol components likely bind free enzyme and the enzyme–substrate complex. The mixed-type inhibitions can arise in many ways: the inhibition of hydrosol could arise because they interact with an afterward intermediate in the reaction pattern but not with an initial enzyme-substrate complex [40]. Thus, we determined dissociation constants (*K_i_* and *αK_i_*, respectively) for the inhibitor binding to the free enzyme (E) and the enzyme–substrate complex using double-reciprocal plots and plots of slope and vertical intercept versus the concentrations of hydrosol in the presence of *L*-tyrosine (Figure 4C,D) or *L*-DOPA as substrates (Figure 4E,F). As shown in Figure 4A,B, the *K_i_* value when *L*-tyrosine was used as the substrate was approximately 1.28 times lower than that in the presence of *L*-DOPA, indicating more effective inhibitory enzyme binding with *L*-tyrosine than with *L*-DOPA. Additionally, the value of *αK_i_* was 1.71 times greater than the *K_i_* value for the oxidation of *L*-DOPA, indicating that the affinity of hydrosol components for free enzyme is stronger than that for the enzyme–substrate complex. These data suggest that hydrosol affects the affinity of the enzyme for *L*-DOPA but does not bind the active site. Moreover, the value of *αK_i_* was almost same as *K_i_* for the oxidation of *L*-tyrosine, indicating that the mixed inhibitors is the noncompetitive inhibitors, which bind to a free enzyme and an enzyme-substrate complex with the same equilibrium constant using L-tyrosine as the substrate (Figure 4A). However, the equilibrium binding constants for the free enzyme and the enzyme-substrate complex, respectively, are different using *L*-DOPA as the substrate (Figure 4B), indicating that a mixed type (competitive and noncompetitive mixed) inhibitor can bind not only with a free enzyme but also with the enzyme-substrate complex using *L*-DOPA as the substrate. Previous studies also revealed that *C. cassia* essential oil and cinnamaldehyde are mixed-type inhibitors [19]. In contrast, *trans*-cinnamaldehyde isolated from the bark of *C. cassia* indicated competitive inhibition for the oxidation of *L*-DOPA by mushroom tyrosinase [32], while cinnamaldehyde isolated from the root of *P. cernua* was a noncompetitive inhibition [35].

### 3.5. Concentration-Dependent Effects of Hydrosol Concentrations on Melanogenesis

**Figure 5:**
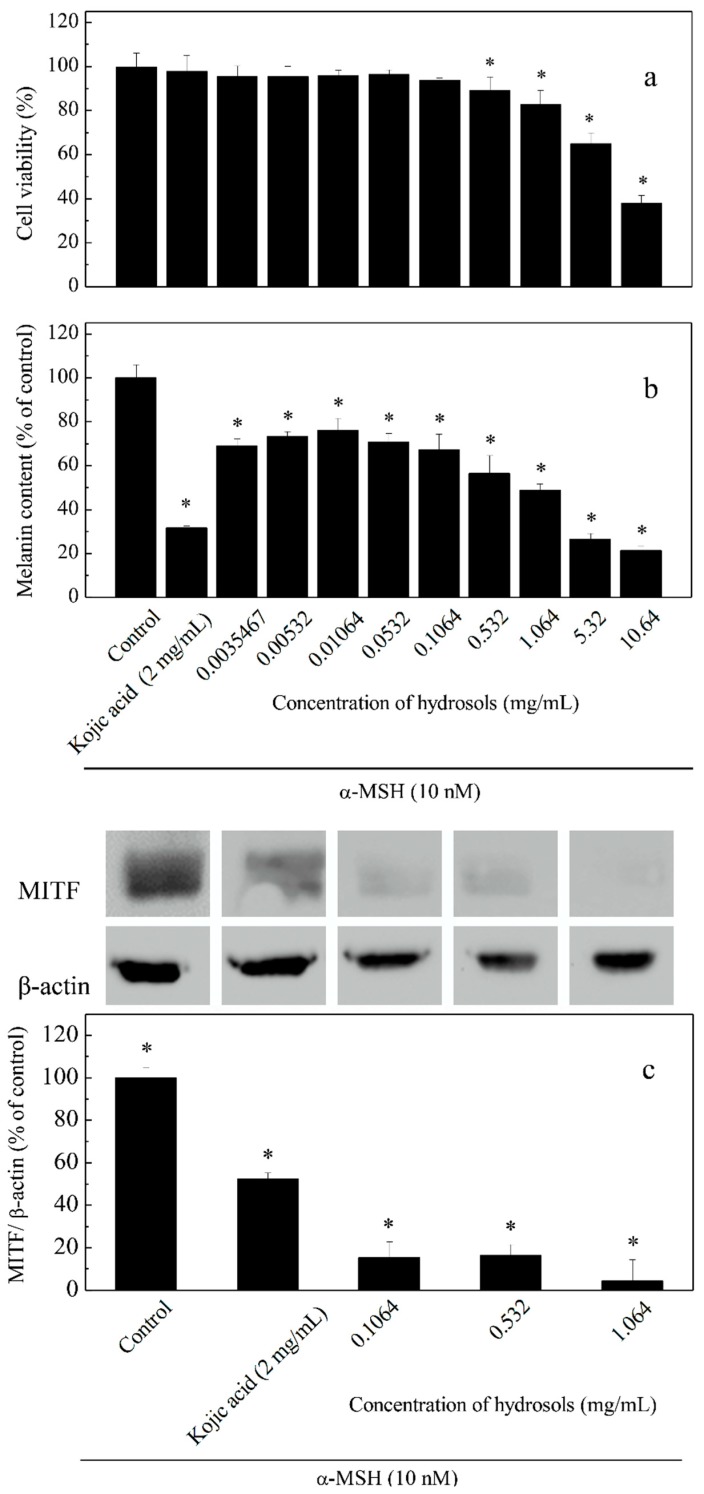
Effects of hydrosol concentrations on cell viability (**a**), melanin contents; (**b**) and expression levels of tyrosinase and microphthalmia-associated transcription factor (MITF); (**c**) in α-melanocyte-stimulating hormone -treated B16-F10 cells. Data are presented as means ± SD from three independent experiments; * *p* < 0.05 compared with the untreated group.

To assay the effects of hydrosol on cell viability and melanogenesis at varying concentrations (0.0035–10.64 mg/mL), we induced melanogenesis in B16-F10 melanoma cells using α-MSH and compared the effects using kojic acid as a positive control (Figure 5). The result of this cell viability assay indicated that hydrosol had no cytotoxicity at concentrations of 0.0035–1.064 mg/mL in B16F10 melanoma cells (Figure 5a). As melanogenesis inhibitors are being developed, safety and effectiveness should be the most important consideration for many applications. However, treatment of B16F10 melanoma cells with hydrosol resulted in a marked reduction of cell viabilities at concentrations of 5.32–10.64 mg/mL. According to results from studies screening of safety and efficacy of skin-whitening products in B16F10 cell cultures, 1.064 mg/mL is a threshold concentration for cell-based primary screening. Therefore, concentrations of 0.1064–1.064 mg/mL were utilized to examine the mechanisms of anti-tryosinase in B16F10 melanoma cells by hydrosol. 

Repression of melanogenesis by hydrosol treatments at various concentrations was determined as percentages of melanin contents in cells (Figure 5b). We also analyzed MITF expression levels using Western blotting (Figure 5c). In these experiments, melanin contents and MITF expression levels were dose-dependently suppressed by COK hydrosol, suggesting that COK hydrosol decreases melanin contents in cells by repressing MITF synthesis.

### 3.6. DNA Protection Assays

**Figure 6:**
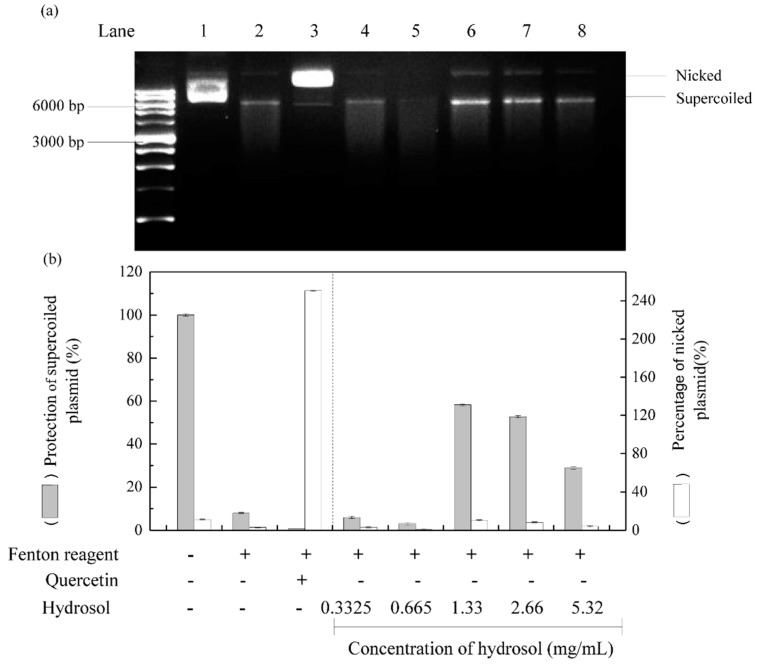
Effects of hydrosol in pCI neo DNA protection assays; (**a**) agarose gel of pCI neo DNA after exposure to Fenton reagents and protective treatments; (**b**) percentage quantification of supercoiled and nicked pCI neo forms; Lane 1, pCI neo DNA, negative control; Lane2, pCI neo + Fenton reagents, negative control; Lane3, pCI neo + Fenton reagent + 250 mg/mL quercetin, positive control; Lanes 4–8, pCI neo + Fenton reagent + various concentrations of hydrosol.

Reactive oxygen species are well known to damage DNA and cause cell ageing and cancer. DNA nicking assays offer a convenient cell-free model system to sensitively determine the production of DNA damaging radicals [41]. In these experiments, Fenton reactions produced hydroxyl radicals that cleave super coiled plasmid DNA and convert it to the nicked form of DNA, which has decreased electrophoretic mobility. [42,43]. Thus, to evaluate DNA protective activities of hydrosol, we incubated *pCI neo* DNA with the Fenton reactants [44]. As shown in Figure 6, supercoiled and nicked plasmid forms are clearly distinguished by their relative electrophoretic mobility rates on agarose gel; supercoiled DNA moved the fastest and nicked DNA moved the slowest, respectively. Following treatments with hydrosol, DNA damage was slightly reduced at hydrosol concentrations of 1.33–5.32 mg/mL, with 28–58% protection of the supercoiled form, respectively (lanes 6–8). At hydrosol concentrations of 0.3325–0.665 mg/mL, no protection was observed relative to the negative control (lanes 4 and 5). In contrast, quercetin effectively protected plasmid DNA from hydroxyl radical-mediated fragmentation, as shown previously [45]. These are the first data to show protective effects of hydrosol extracts from *C. osmophloeum* Kanehira leaves on DNA damage by Fenton reactions. These effects are likely due to the presence of polyphenol compounds.

## 4. Conclusions

COK has long been used as a medicinal plant in Taiwan. However, to our knowledge, this is the first report demonstrating that hydrosols that are prepared by steam distillation from COK leaves have antioxidant activities, as observed in assays of antioxidant activity and DNA damage. We also showed that this hydrosol represses melanogenesis. In GC/MS analyses, the major compounds in COK hydrosol were found to be cinnamaldehyde and benzaldehyde, and these agents were potently inhibitory against the monophenolase and diphenolase activities of tyrosinase. In our assessments of tyrosinase inhibition kinetics, COK hydrosol exhibited mixed-type dose-dependent inhibition of both *L*-tyrosine and *L*-DOPA oxidase activities of tyrosinase. The present hydrosol compounds also suppressed MITF protein expression, leading to reduced α-MSH-induced melanin synthesis.

Finally, safety is a major consideration for tyrosinase inhibitors, especially for those used in cosmetic and food products, which may be used in regulated quantities. COK is already an edible and extensively used natural additive for food and cosmetics. Furthermore, cinnamaldehyde is generally recognized as safe for human consumption. COK hydrosol is an excellent natural biomaterial with effective and safe tyrosinase and melanin synthesis inhibitory activity and potential to protect against DNA damage. Therefore, we believe that COK hydrosol could be used as a safety and effective depigmenting agent for many applications.

## Figures and Tables

**Table 1:** Summary of the tyrosinase inhibitory activity of compounds from natural sources.

Compounds	Type of Inhibition	IC_50_ (mM)	IC50 (mg/mL)	References
kojic acid	mixed-type ^b^	0.014	0.002	[29]
Anisaldehyde	Non-competitive ^b^	0.32	0.044	[31]
Anisaldehyde	Non-competitive ^b^	0.38	0.052	[32]
Benzaldehyde	Non-competitive ^b^	0.83	0.088	[32]
Cuminaldehyde	Non-competitive ^b^	0.05	0.007	[31]
Cinnamaldehyde	Non-competitive ^b^	0.98	0.145	[33]
Cinnamaldehyde	Non-competitive	4.73	0.701	[34]
Cinnamaldehyde	Noncompetitive	0.97	0.144	[35]
Eugenol	Competitive	12.83	2.107	[34]
Trans-cinnamaldehyde	Mixed-type ^b^	4.04	0.534	[19]
Trans-cinnamaldehyde	Mixed-type ^a^	0.0053	0.0007	This study
Trans-cinnamaldehyde	Mixed-type ^b^	2.32	0.307	This study
Trans-cinnamaldehyde	Competitive ^b^	0.85	0.112	[32]
Flavanols				
(–)-Epigallocatechin	Competitive ^a^	0.035	0.011	[4]
(–)-Epicatechin gallate	Competitive ^a^	0.017	0.008	[4]
(–)-Epigallocatechin gallate	Competitive ^a^	0.034	0.015	[4]
Flavonols				
Quercetin	Competitive ^b^	0.07	0.024	[36]
Kaempferol	Competitive ^b^	0.23	0.066	[36]
Morin	Competitive ^b^	2.32	0.701	[36]
Flavones				
Luteolin	Non-competitive ^b^	0.19	0.054	[36]
Luteoilin 7-O-glucoside	Non-competitive ^b^	0.5	0.143	[36]
Isoflavans				
Glabridin	Non-competitive ^b^	0.004	0.001	[37]
Glabrene	Mixed-type ^b^	7.6	2.450	[37]
Isoliquiritigenin	Mixed-type ^b^	0.047	0.012	[37]

^a^ Inhibition of monophenolase activity (using L-tyrosine as the substrate). ^b^ Inhibition of diphenolase activity (using L-DOPA as the substrate). IC_50_, inhibitory concentration 50%.
